# Which Role Model Is More Effective in Entrepreneurship Education? An Investigation of Storytelling on Individual’s Entrepreneurial Intention

**DOI:** 10.3389/fpsyg.2019.00837

**Published:** 2019-04-24

**Authors:** Feng Liu, Jiangshui Ma, Ran Li

**Affiliations:** ^1^School of Public Affairs and Law, Southwest Jiaotong University, Chengdu, China; ^2^School of Business Administration, Southwestern University of Finance and Economics, Chengdu, China

**Keywords:** entrepreneurship education, role model story, entrepreneurial intention, entrepreneurial passion, self-efficacy

## Abstract

Entrepreneurial stories, such as the breathtaking experience of Steve Jobs, are often adopted as an effective teaching instrument to promote individuals’ entrepreneurial intentions in entrepreneurship education. Prior research highlights the role model’s influence and the positive effect of entrepreneurial stories, which is taken for granted in many circumstances. Since most research has treated storytelling in teaching as an undifferentiated whole, few researchers have teased out the distinctive effect of different types of entrepreneurial stories, namely that between successful stories and failure stories, and between idol stories and peer stories. To deepen our knowledge about how distinctive entrepreneurial stories affect entrepreneurial intentions, we conducted two experimental studies on 150 undergraduate students in entrepreneurship education programs (EEPs). Results show that, through the intermediary variable of entrepreneurial passion, both successful stories and failure stories positively influence entrepreneurial intentions as educators presumed, but successful role model stories have a greater impact than failure stories. While idol stories, rather than peer stories, are more inclined to arouse individuals’ entrepreneurial intentions. Furthermore, we find that individuals with low entrepreneurial self-efficacy are less affected by the storytelling process.

## Introduction

In the past decades, entrepreneurship has become increasingly prominent in reducing youth unemployment, improving financial stability and promoting economic development (Bohlmann et al., 2017). As a critical role in social development, entrepreneurship not only takes academic scholars’ notice, but also attracts many government officers’ and educators’ attentions. In order to inspire entrepreneurship activities, governments and scholars have both made great efforts to assist nascent entrepreneurs, such as establishing incubators, introducing supportive policies and setting up entrepreneurship education programs (EEPs). Such undertakings have been based on the implicit premise that these efforts will inspire entrepreneurial spirit (Obschonka et al., 2018), enhance nascent entrepreneur’s self-efficacy and promote entrepreneurship motivation (Syam et al., 2018). Indeed, many studies show that entrepreneurship education contributes to people’s entrepreneurial intentions and reinforces their entrepreneurial activities.

Even though many studies have shown the positive relationship between entrepreneurship education and entrepreneurial intention, there is no consensus on what and how entrepreneurship education actually influences people in practice (Pittaway and Cope, 2007). Besides, some scholars like Oosterbeek et al. (2010) find that entrepreneurship education is negatively related to entrepreneurial intention. A critical reason for this contradictory notion is that many scholars treated entrepreneurship education as an undifferentiated whole, ignoring the variety in content and pedagogy in different EEPs (Piperopoulos and Dimov, 2015). It warrants a closer look at the entrepreneurship education mechanisms, especially the popular content, such as Steve Jobs’ entrepreneurial story, which is widely taught in college curriculums. Drawing upon the extensive use of storytelling in entrepreneurship education, we provide a deeper investigation of different types of entrepreneurial stories in this paper, arguing that the effects of different role models vary extensively.

Using metric conjoint analysis, we tested our hypotheses in two experiments through 150 undergraduate students who enrolled in a 6-week EEP. The two experiments, which were carried out in a Chinese university, demonstrated that through the intermediary variable of entrepreneurial passion, both successful stories and failure stories have positive impacts on entrepreneurial intentions. However, the successful role model stories have a greater impact than failure stories. Furthermore, rather than idol stories, which are frequently utilized by educators worldwide, peer stories are more inclined to arouse individuals’ entrepreneurial intentions. In addition, we find self-efficacy moderates the storytelling process, whereas audiences with low entrepreneurial self-efficacy are less affected by entrepreneurial stories.

The paper is organized as follows. In the first section, we raise the question and discuss the theory background. We present the status of current research which relates to the evaluation and impact of case-based teaching in EEPs and, in a broader sense, on the antecedents of entrepreneurial intention as studied in the literature. The second section is devoted to our theoretical and methodological approaches, while the third section describes the samples and research experiments utilized in this paper. The following sections outline and discuss our results. We also look into the limitations of our work. In conclusion, we underline our main findings, derive their theoretical and practical implications, and then present possible directions for future research.

### Theoretical Development

According to the Global Entrepreneurship Monitor (GEM) 2015/2016 Report, China’s entrepreneurial activities index was 12.84%, which was relatively high compared with other countries in the G20, such as the United States (11.88%), the United Kingdom (6.93%), Germany (4.70%), and Japan (3.83%). However, only 2.93% of Chinese college graduates chose to undertake entrepreneurial activities in 2016, whereas this ratio is up to 20–23% in United States^1^. Such a remarkable disparity motivated us to consider the entrepreneurship education in China, especially the teaching process in universities. Why does the entrepreneurship education in China not achieve the same outcome, of arousing entrepreneurial intention, as well as other countries?

With this question in mind, researchers have explored diverse factors in determining entrepreneurial participation, including individual factors, such as demographics (e.g., experience in some area), behavioral (e.g., management style), psychological and/or personality (e.g., risk propensity and dark triad) cognitive traits (e.g., entrepreneurial alertness) (Busenitz and Barney, 1997; Miner, 2000; Shane and Venkataraman, 2000; Gaglio and Katz, 2001; Hu et al., 2018; Wu et al., 2019), and external environment factors (Lee et al., 2011; Alessandro and Vita, 2016). In the literature, SCT (social cognition theory) is a commonly used tool for analyzing the impact of external and personal factors on entrepreneurial intentions. Planned behavior theory (Ajzen, 1991) and SCT are probably the most widely used models in prior literature, improving our understanding of the entrepreneurial intentions (Autio et al., 1997; Tkachev and Kolvereid, 1999; Tounés, 2003; Audet, 2004; Liñán, 2004; Boissin and Emin, 2006; Fayolle and Gailly, 2015; Biraglia and Kadile, 2017).

In the same stream, this paper deepens our understanding of this question by exploring the different effects of various role models in the storytelling process and investigating the entrepreneurship education by examining the individual factors and environmental factors jointly. Although storytelling methodology in case-based teaching is a popular choice, limited scholars test whether different types of role models influence similarly or not. The main purpose of this paper is to investigate the entrepreneurial story’s influence process, providing an understanding of which entrepreneurial stories take effect through EEPs, and how. We teased out different types of entrepreneurial stories, namely between successful stories and failure stories, and between idol stories and peer stories. In addition, we scrutinized the storytelling’s dynamic influencing mechanism by inspecting the mediating role of entrepreneurial passion and the moderating role of audiences’ self-efficacy.

### Entrepreneurship Education

As both governments and scholars esteem entrepreneurship activities highly, EEPs are exploding across the world, from a handful of universities in the 1970s to the majority of higher education institutions (Katz, 2003). More than 2,200 courses are offered at over 1,600 schools in United States (Kuratko, 2005). According to Fayolle and Gailly (2008), entrepreneurship education fosters entrepreneurial attitudes, skills and mind-set. The principal role of an EEP is to promote students’ entrepreneurial intentions and increase their awareness that the entrepreneurial path is a viable career option (Fayolle and Gailly, 2015).

An abundance of prior research has found that entrepreneurship education impacts entrepreneurial intentions positively (Chen et al., 1998; Zellweger et al., 2011), but the effectiveness and validity of entrepreneurship education is still unclear, as entrepreneurship education is often treated as a single monolith. Given the widely accepted notion that entrepreneurship education is critical for venture creation, relevant questions as regards teaching content and methodologies are brought up: What should be taught and how should it be taught (Kuratko, 2005)? Indeed, a lot of research have been done through various perspectives, from entrepreneurship education evaluation to program implementation. For instance, scholars have provided valuable insights into pedagogies for entrepreneurship education. Solomon et al. (2002) investigated methodologies adopted in the daily teaching curriculums such as experiential learning, business plans, computer simulations, field trips and the use of video and films, and technology application. Whether students are benefited or not relies on the involvement and participation throughout the teaching process (Yar Hamidi et al., 2008).

### Storytelling in Entrepreneurship Education

Entrepreneurial stories inspire people’s entrepreneurial intentions and encourage the process of emulation (Bouwen and Steyaert, 1997; Laviolette et al., 2012). Storytelling in the curriculums is not only about learning knowledge and skills, but also about developing nascent entrepreneurs’ future-oriented imagination and influencing their career choices. Lots of research verifies that EEP is an important methodology to foster entrepreneurial intention (Peterman and Kennedy, 2003; Beugelsdijk et al., 2004). Case-based teaching is frequently adopted as an effective teaching method in EEPs by telling entrepreneurial role model stories, such as those of Steve Jobs, Jack Ma, and Zuckerberg’s experiences. After editing and rewriting, their stories are compiled into Harvard or Ivey Cases. Moreover, universities also tried to invite the protagonists of each case to the classrooms in order to present the entrepreneurial stories vividly.

In comparison, little attention has been devoted to investigating the different types of entrepreneurial stories, and no clue is provided for whether different types of role models affect similarly or not. What remains to be verified is whether the positive effect is still robust across the different types of content and pedagogies that are utilized in EEPs. Compared with the consistent agreement that observing successful entrepreneurs can enhance the entrepreneurial intentions (Fellnhofer, 2017), limited studies emphasize the effect of telling failure stories in the teaching process, and scholars hold different views on this. Many studies argue that both successful stories and failure stories facilitate individuals’ entrepreneurial intentions (Minniti and Bygrave, 2001; Lockwood et al., 2004), as long as failure is the mother of success.

However, some scholars argue that failure stories may be more effective when the aim is to avoid an undesirable state or result (Laviolette et al., 2012). For instance, failure stories may attenuate audiences’ intentions by signaling to them that entrepreneurship requires more than they expected (Oosterbeek et al., 2010). Will failure peer stories raise audiences’ caution for risk and diminish their motivations? Such questions are still ambiguous and imprecise, inviting more substantive categorizations for role models and separate investigations into them (Piperopoulos and Dimov, 2015). In this paper, we categorized entrepreneurial stories by two dimensions: successfulness and distance, and four groups of stories are discussed.

### Hypotheses

Role Models, defined as those who can encourage others to pursue certain career paths or pursue certain goals (Basow and Howe, 1980), are adopted widely in the field of entrepreneurship education. Heroes like Washington and Napoleon always inspire youths worldwide. Through the role model’s experience, individuals may subconsciously develop their mentality, imitate the role model (Biraglia and Kadile, 2017), and strive to become the role model (Laviolette et al., 2012). Krumboltz et al. (1976) suggested that role models have a profound impact on individual’s career choices and this proposition was affirmed by other scholars (Krueger et al., 2000; Douglas and Shepherd, 2002). Shapero and Sokol (1982) first introduced the concept of role models to entrepreneurial research and predicated that family members, especially parents, would affect individuals’ perceptions as entrepreneurial role models. Narrative and storytelling can potentially influence people’s entrepreneurial decisions through knowledge sharing and expression of experiences (Ajzen, 1991; Akerlof and Kranton, 2000; Fellnhofer, 2017).

Undoubtedly, role model education is adopted as a useful teaching instrument in EEPs (European Commission, 2003; Organisation for Economic Co-operation and Development, 2009). A lot of scholars believed that successful role models have positive impacts on entrepreneurial intentions because individuals are motivated by imagining their own achievements as that of a successful role model in the future (Taylor et al., 1996; Aspinwall, 1997; Lockwood and Kunda, 1997, 1999; Chang, 2014). One of the most important incentives is that educators believe role model’s stories will arouse audiences’ entrepreneurship spirit (Krueger et al., 2000; Van Auken et al., 2006; Bosma and Schutjens, 2011), while many of them propose that this influence is conditional on whether the role model’s achievement is achievable for the audiences (Lockwood and Kunda, 1997; Lockwood and Kunda, 2000; Blanton, 2001).

Even though role model education is warmly embraced by educators and students, and covers most entrepreneurship curriculums in universities, some scholars possess different opinions and express mixed comments, especially for failure role models. Compared with successful role models, conclusions for failure role models are much more controversial, and the literature provides mixed results. On the one hand, some studies suggest that the incomplete and ambiguous failure information will raise nascent entrepreneurs’ anxiety of entrepreneurial risk and attenuate their passions. On the other hand, failure role models are valuable to entrepreneurs by strengthening their sensitivity and avoiding potential failure risks (Lockwood et al., 2002; Minniti and Nardone, 2007).

With such conflicting accounts regarding the impact of role model stories, many scholars attempted to resolve this debate from different perspectives. In particular, Lockwood et al. (2002) argued that prior studies have not taken into account that the effect of role models may depend on the goals which audiences intend to pursue. As promotional individuals are more sensitive to successful role models, failure role models have more influence on audiences with prevention goals. Bandura (2010) proposed that self-efficacy plays an important role in an individual’s achievement of goals since people with high self-efficacy have more courage to try new things. Moreover, based on the SCT frame work, Bandura (1989) suggested that passion is critical in the role model teaching process. Drawing upon the stream of SCT literature, Geneve et al. (2008) illustrated how women can maintain the passion for the DCI (Digital Content Industry) profession through alternative learning acquired from role models. For the literature review presented above, we propose the following research hypotheses in the rest of this section and detail the theoretical framework.

### Role Model Stories and Entrepreneurial Intention

Role model stories in EEPs not only provide spiritual incentives for audiences, but also offer behavioral guidance for potential entrepreneurs (Gibson, 2004; Bosma et al., 2012). Through spiritual incentives and behavioral guidance, role model stories influence entrepreneurial behaviors in the process of people’s career choices (Shapero and Sokol, 1982; Brenner et al., 1991; Lent et al., 1994). Particularly, role model stories can stimulate entrepreneurial intention by providing positive information (Gnyawali and Fogel, 1994) and reference information (Minniti and Nardone, 2007). This information in the role model stories helps individuals to discover entrepreneurial opportunities and reduce risks in the future venture creation process. Depending on the external environment and individual factors, these stories consolidate individuals’ entrepreneurial intentions (Stapel and Koomen, 2001; Lockwood, 2006).

Hence, the following research hypothesis was postulated in the present study:

**Hypothesis 1:** Role model stories have a positive impact on entrepreneurial intentions.

### Success/Failure Stories and Entrepreneurial Intention

According to the expectations theory, positive outcomes lead to an increase in the expectation of good results, while negative outcomes have a weakening effect. Individuals enjoy scrutinizing successful entrepreneurship models, which potentially enhances their original entrepreneurial intention (Fellnhofer and Puumalainen, 2017). Comparing this with the agreement on successful role model stories (Chang, 2014), recent entrepreneurship studies cast some doubt about the specific influence of failure role model stories. From the expectation theory, failure role model stories reduce individual’s expectations of the event. However, McGrath (1999) find that failure role models influence potential entrepreneurs positively. Given the finds in previous research, we propose that failure role models stories also influence entrepreneurial intentions positively, but less so than successful role model stories.

Hence, the following research hypothesis was postulated in the present study:

**Hypothesis 2:** Successful role model stories have a greater impact on entrepreneurial intentions than failure role model stories.

### The Mediating Role of Entrepreneurial Passion

From the SCT perspective (Bandura, 1986), external environment, personal factors and individual intentions interact with each other dynamically. Mehrabian and Russell (1974) concluded that the external environment’s stimulation of behavior must be influenced through emotional responses. Following this guideline, we argue that external environment influences people’s intentions through individual factors. More specifically, role model stories influence entrepreneurial intentions through emotional responses. However, the mediating role of the personal factor is not mentioned in many related studies. With regard to the limitation of current conceptualizations of the relationship between the entrepreneurial role model and career choice, Barnir et al. (2011) proposed the possibility of intervening variables which may complement the literature deficient. Their suggestion corresponds to the passion theories, that domain-specific work passion drives motivation and engagement due to its motivational effect (Obschonka et al., 2018). People may generate different emotional beliefs for the same role model story, bringing various conclusions and distinctive entrepreneurial intentions. Consistent with this viewpoint, Obschonka et al. (2018) investigated the mediating effect of entrepreneurial passion between personality and entrepreneurial behavior.

Passion, as an important personal factor for entrepreneurship (Baum et al., 2001), is considered to be a relatively stable personality trait and drive people to react consistently in different situations (Chu, 2011). Tremendous research has explored entrepreneurial passion and its effectiveness (Cardon, 2008; Cardon et al., 2013; Cardon and Kirk, 2015). However, the issue of entrepreneurial passion as a factor for entrepreneurial intention has not yet been revealed in the literature. Whether entrepreneurial passion is a key factor in the potential entrepreneurial intention is worth exploring and verifying (Fellnhofer and Puumalainen, 2017). Deeper examination for the impact of entrepreneurial passion is urgent in this area (Gibson, 2004; Stam and Schutjens, 2004).

Hence, the following research hypothesis was postulated in the present study:

**Hypothesis 3:** Role models stories have a positive impact on entrepreneurial intentions through the intermediary variable of entrepreneurial passion.

### The Moderating Effect of Entrepreneurial Self-Efficacy

Self-efficacy, defined as a person’s belief in her/his own ability to achieve a goal (Bandura, 1977), is widely applied to entrepreneurial intention within the field of entrepreneurship (Lent and Hackett, 1987; Krueger et al., 2000; Sequeira et al., 2007). Lee et al. (2011) found that the self-efficacy works as a moderator in the relationship between perceived desirable and entrepreneurial intentions. When facing low job satisfaction, individuals with high self-efficacy are more willing to start a business. Recent research shows that self-efficacy is positively related to many entrepreneurial behaviors. For instance, Chen et al. (1998) found that entrepreneurs possess higher self-efficacy than managers. Hmieleski and Corbett (2008) revealed the positive relationship between self-efficacy and new venture performance. In this study, we concentrate on task-specific self-efficacy, rather than general self-efficacy, since task-specific self-efficacy is more predictive when studying entrepreneurial behaviors (Chen et al., 1998).

Focusing on entrepreneurship education, we argue that individuals with different self-efficacy engender distinctive reactions for the role model stories. More specifically, successful role model stories are more likely to impress audiences with high self-efficacy and failure role model stories are more likely to impress audiences with low self-efficacy. Since empirical studies rarely have examined the moderation effect of self-efficacy (Lin and Si, 2014), this study verifies entrepreneurial self-efficacy’s moderating effect. We propose that individuals with high self-efficacy are more sensitive to entrepreneurial role model stories.

Hence, the following research hypothesis was postulated in the present study:

**Hypothesis 4:** Entrepreneurial self-efficacy plays a positive role in the relationship between entrepreneurial role models’ stories and individuals’ entrepreneurial intentions. When students’ self-efficacy is high, the relationship will be stronger.

### The Moderating Effect of the Distance Between Audience and Role Model

Besides personal factors, many researchers have emphasized the influence of environmental factors on entrepreneurial intention (Fayolle and Gailly, 2015). Hite and Hesterly (2001) believed that a close relationship with the role model has a stronger influence on an individual’s entrepreneurial intention. Therefore, the relationship between success or failure role models and entrepreneurial intention may be affected by the distance between the audiences and role models. The strength of the relationship depends on the ability to make frequent or accessible connections. For example, children’s attitudes toward entrepreneurship are affected by their family members who operate businesses (Carr and Sequeira, 2007), and peers influence individual’s entrepreneurial activities among MBA students (Malmendier and Lerner, 2007). Nevertheless, the influence of entrepreneurship models comes from various aspects, not only face-to-face communication but also through the media, magazines and the internet which are far away. Thus, in entrepreneurial education, real contact between role models and the audience is not essential; role models may be proximal individuals, or can be celebrities, fictional characters, or historical figures. Social comparison does not require personal contact but rather identification and motivation to become “like the other” (Wilson et al., 2007).

In this paper, we utilize the distance between audience and role models as criteria and test its moderating effect on the relationship between storytelling and entrepreneurial intentions. Hite (2010) described the relationship between individuals and entrepreneurship as a strong tie or weak tie, and found the stronger tie has a greater impact on individual’s entrepreneurial intention in the early stage of entrepreneurship. Peterman and Kennedy (2003) even ranked the role models by the influence process: family members were the most influential models, followed by peers, and idols. To correspond with this, we suggest that the distance between role model and audience is an important factor for storytelling influence process, and hypothesize the following:

**Hypothesis 5:** The distance between role model and audience influences the relationship between a success or failure story and audience’s entrepreneurial intention.

**Hypothesis 6:** Peer role models have a stronger impact on the relationship between the success or failure story and audience’s entrepreneurial intention than idol role models.

## Methodology

Before presenting the sample and collection processing of the data, we give a brief overview of the characteristics of the EEPs under examination here. Two experiments are conducted in the EEPs to explore the validation of our hypotheses.

**FIGURE 1:**
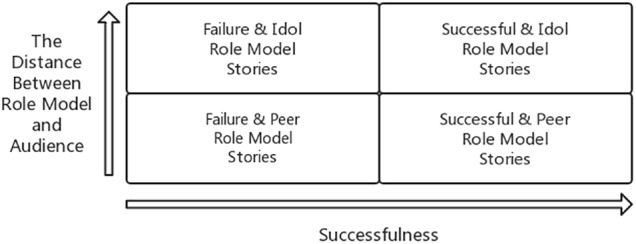
The types of entrepreneurial story.

**FIGURE 2:**
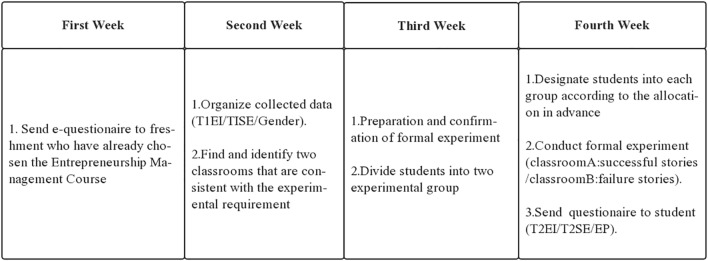
The flow of experiment 1.

This study explores the impact of the story-based teaching in EEPs through metric conjoint analysis. The conjoint analysis, which uses a series of profiles to capture participants’ assessments are collectively used to decompose the underlying structure of respondents’ decisions (Shepherd and Zacharakis, 1999). Conjoint analysis explores the judgments of potential entrepreneurs on whether to conduct entrepreneurship and overcome the shortcomings of other research designs, therefore, it’s a good choice in studying the effect of storytelling (Scheaf et al., 2018). In addition, this study uses a 2 × 2 hybrid design with self-variables for the class (experimental and control groups) by between-subjects design, and the measurement phase (pre-test and post-test) using the subjects (within-subjects design). The experiment process is shown in Figure 1 while Figure 2 summarizes the hypothesized relationships and the research design.

Investigating entrepreneurs’ psychology is challenging, in part, because psychological variables and causal mechanisms are unobservable (Dan et al., 2017). The experimental method is an effective tool in confirming the causal relationships since it offers good control (reducing or eliminating the possible unrelated factors according to the research purpose), active transformation (actively manipulate experimental conditions to meet the needs of the experiment), and more realistic and effective data. As entrepreneurial intention, entrepreneurial passion, and self-efficacy are individual’s psychological factors we employ the experimental method in this research. The controllable classroom provides a suitable environment condition to explore the role model’s storytelling influence mechanism.

The EEPs named Entrepreneurship Management Course that we have chosen for Study 1 and Study 2 spanned 25 h over 6 weeks. The EEPs in our two experiments have the same content but different participants. Considering that freshmen are relatively naive and lack basic perception of business, the EEPs may be considered as an entrepreneurial awareness program. From a content point of view, the EEP was designed to provide practical guidance by covering a range of entrepreneurial knowledge, including basic concepts, initial mental preparation, opportunity identification and creation, innovation and transformation, and business launching. Issues about self-awareness, identifying opportunities, creative thinking, developing business models, entrepreneurship laws, BPs and securing external funding are all mentioned and discussed.

Entrepreneurship education programs for Study 1 was attended by 74 new students (37 males and 37 females) while EEP for study 2 was attended by 76 students (38 female and 38 male) from various bachelor disciplines in Southwest Jiaotong University in China. Both studies were divided into two parts: *t* pre-test and post-test. Measurements of dependent and independent variables in our model were based on a questionnaire administered to all participants at three different time periods: beginning of the program, immediately after the storytelling course, and after all courses completed.

These questionnaires are directly inspired by those developed and validated by Kolvereid (1996a,b). The three questionnaires in both experiments all included questions related to the measurement of the parameters of Ajzen’s intention model and Cardon’s entrepreneurial passion scale, including 13 Likert-scale items (with scores ranging from 1- “Totally disagree” to 7- “Totally agree”). In the questionnaires, six items concerned self-efficacy (for instance “I have the ability to solve problems”), three items concerned entrepreneurial passion (for instance “establishing a new company excites me”), and four items concerned entrepreneurial intention (for instance “I already have a business plan.”) (see Appendix: Variable Measurement).

The first questionnaire also included a specific part with participants’ background information concerning socio-demographic variables (Robinson et al., 1991) and other variables related to prior entrepreneurial exposure and experience. Therefore, it allowed us to measure control variables (gender, initial self-efficacy, and initial entrepreneurial intention) in our model. All three questionnaires were administered in the same conditions. A specify member of the research team was designated to answer potential questions and ensure the operation ran smoothly each time (see original data in the Supplementary Materials).

## Measures

### Independent Variable

Successful entrepreneurial story and failure entrepreneurial story were used as independent variables in both experiments, the successful entrepreneurial story was coded as 1, and the failure entrepreneurial story was coded as 0.

### Dependent Variable

Entrepreneurial intention is the dependent variable in current research and the measurement mainly adopts the continuous measurement method of multiple items, among which the four-item scales of Zhao et al. (2005), and the five items of Chen et al. (1998) were most commonly used. In this study, we chose Chen’s method to measure entrepreneurial intention. As freshmen have no concept for the question, “When will you set up a new business in the future?”, we deleted it from the questionnaire and retested the questionnaires validity (Cronbach’s alphas = 0.826).

### Control Variable

Some scholars believed that the entrepreneurial intentions for individuals in different stages are highly correlated (Zhao et al., 2005), therefore, this study takes the original entrepreneurial intention as a control variable. Similarly, students’ original sense of self-efficacy was employed as another control variable. In addition, gender is also an important variable that affects the role of entrepreneurial stories in individual entrepreneurial intentions (Laviolette et al., 2012). Therefore, this study also took gender as a control variable.

### Moderator Variable

In study 1, self-efficacy after the experiment was used as a moderator, and the measurement was the same as used for the original self-efficacy beforehand. In study 2, the distance between students and the entrepreneurial role model was employed as a moderator as well. The idol was coded as 0 and the peer was coded as 1 (0 = idol, 1 = peer).

### Mediator Variable

In study 1, we tried to explore the relationship between entrepreneurial stories and a student’s entrepreneurial intentions, and entrepreneurial passion was considered as a mediator between these two variables. There were two popular ways to measure entrepreneurial passion. Vallerand and Houlfort (2003) compiled a scale of entrepreneurial passion from two dimensions: forced passion and harmonious passion. In contrast, Cardon et al. (2009) measured entrepreneurial passions based on the different identities of entrepreneurs from three aspects: innovation passion, creation passion, and development passion. Since all participants in this study are students and they have not had entrepreneurial experience, we used the creative passion dimension to measure entrepreneurial passion. Considering that freshmen may have no concept of actual entrepreneurial behavior, we deleted the item: “Is being the founder of a business is an important part of who I am?”, then retested the questionnaires validity (Cronbach’s alphas = 0.807).

### Experiments Design

**FIGURE 3:**
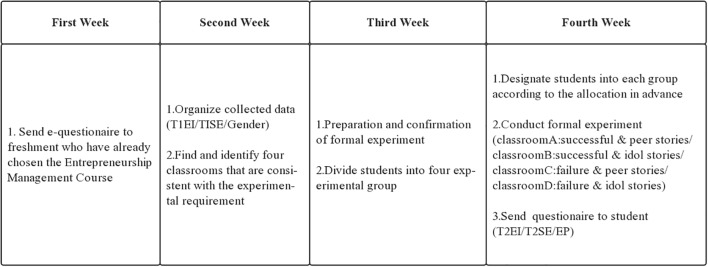
The flow of experiment 2.

Procedures for the two experiments are presented in Figure 2, 3.

**FIGURE 4:**
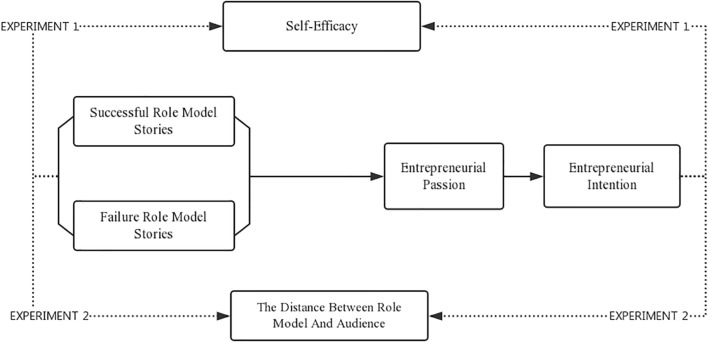
The model of experiments.

Study 1 seeks to: (1) verify the different impact of successful/failed stories on students’ entrepreneurial intention; (2) verify the mediating role of entrepreneurial passion; (3) verify whether entrepreneurial self-efficacy moderates the relationship between role model stories and entrepreneurial intention. Experiment 1 model is shown in Figure 4.

Study 2 seeks to: (1) Re-validate the impact of successful/failure entrepreneurial role model stories on students’ entrepreneurial intentions; (2) Re-validate the different effect of successful and failure entrepreneurial role model stories; (3) explore the moderating effect of the distance between students and role models. Experiment 2 model is shown in Figure 4.

Both experiments were divided into pre-test and post-test and, to collect data, students filled out the questionnaires 1 month before and after the experiment.

### Pre-test

Before the new semester started, an e-questionnaire was sent to freshmen who had already chosen the Entrepreneurship Management Course. The questionnaire mixed the items of entrepreneurial self-efficacy and entrepreneurial intentions into other items about the students’ requirements and expectations for the course. In this way, we hoped to achieve the purpose of covering up the experimental intentions. Three data, including student’s original entrepreneurial intention (T1EI), original self-efficacy (T1SE), and gender, were collected.

### Formal Experiment

In study 1, the teacher informed students that the class was divided into two groups for discussion. According to the random allocation in advance, 37 students were designated to each group. One group stayed in Classroom A and one group went to pre-prepared Classroom B. Students in Classroom A were told a successful entrepreneurship story while students in Classroom B were told a failure story. After storytelling, each group had a discussion with their storytellers to get more information (see Figure 1). In study 2, before the course began, students were divided into four groups and assigned separately to classroom A, B, C, and D. The four groups took part in the experiments with almost identical conditions and heard different entrepreneurial role model stories (see Figure 3). Also, after storytelling, each group had a discussion with their storytellers to gain more information.

### Post-test

After students finished the experiment, teachers issued a questionnaire to them, seeking to understand their feelings and evaluations. The questionnaire also mixed the items related to entrepreneurial intention (T2EI), self-efficacy (T2ES), and entrepreneurial passion with other items about evaluating the course. Items related to entrepreneurial intention and entrepreneurial self-efficacy were changed in expression, and, as pretest was conducted 1 month before the start of the course, the likelihood of exposure for experimental purposes was reduced.

## Analysis and Results

### Result for Study 1

Appendix Table A1 reports descriptive statistics for study 1. Students’ original entrepreneurial intention (2.9196) is lower than post-experimental entrepreneurial intention (3.8678), indicating that entrepreneurial intention was generally improved by the storytelling course. Entrepreneurial intention for students that heard successful entrepreneurial stories (4.2265) is higher than that for students who heard failure entrepreneurial stories (3.5091); Entrepreneurial intention for students who heard successful entrepreneurial stories has increased 1.3319, which is higher than students that heard failure stories (0.5644).

Results of hierarchical linear regression to test hypotheses were showed in Appendix Table A2. In Model 1, we examined the baseline model with only control variables: gender, entrepreneurial self-efficacy. Results show that original entrepreneurial intention (T1) (β = -0.112, *p* > 0.05) has no significant influence. But gender (β = 0.338, *p* < 0.01) and self-efficacy (T1) (β = 0.296, *p* < 0.01) before the experiment have a significant impact on students’ entrepreneurial intentions (T2) after the experiment.

After independent variables and moderating variables added in Model 2, the influence of the control variables on the entrepreneurial intention (T2) disappeared. The positive and significant effect (β = 0.33, *p* < 0.01) strongly support hypothesis 1. At the same time, the coefficient for successful stories is higher than failure stories. Therefore, hypothesis 2 was supported.

Model 3 added the interaction term between independent variable and moderating variable based on model 2. The positive and significant effect for entrepreneurial stories (β = 0.191, *p* < 0.01) proves hypothesis 1 again. In addition, the interaction effect for self-efficacy (β = 0.388, *p* < 0.05) is also positive and significant and hypothesis 4 is supported. The coefficient for self-efficacy (β = 0.406, *p* < 0.01) is no longer significant after interaction item is added in model 3, suggesting the direct effect of self-efficacy is replaced by its moderating effect. When students’ self-efficacy is high, the impact of stories on students’ entrepreneurial intention will be stronger. Comparing *R*^2^ for the three models, model 3 (β = 0.651, *p* < 0.005) is the best to explain the research problem. Based on the above analysis, hypotheses 1, 2, and 4 are supported.

According to the sequential test of mediator variables (Wen et al., 2004), we examine the mediating effect of entrepreneurial passion as shown in Appendix Table A3. Model 1 tests the relationship between entrepreneurial stories and entrepreneurial intention again where coefficient β = 0.511, (*p* < 0.01); In Model 2, success/failure stories are used as an independent variable, and entrepreneurial passion as a dependent variable. The positive has a significant effect β = 0.0.527 (*p* < 0.01) supporting the relationship between these two variables; In Model 3, the positive and significant coefficient β = 0.542 (*p* < 0.01) supports the relationship between entrepreneurial passion and entrepreneurial intention (T2); Model 4 tests the effect of entrepreneurial passion and entrepreneurial stories on entrepreneurial intention. The influence for entrepreneurial passion (β = 0.377, *p* < 0.01), and the influence for entrepreneurial stories (β = 0.313, *p* < 0.01) are both positive and significant. Therefore, the intermediating role for entrepreneurial passion is proved and hypothesis 3 is supported.

Above, hypothesis 1, 2, 3, and 4 are supported.

### Result for Study 2

Before the experiment, students’ entrepreneurial intentions are similar and the average number is roughly equal to 3.2, however, after the experiment, students’ entrepreneurial intentions are obviously different. Average growth rate of entrepreneurial intention for students who listened to the failure-peer story is 0.6339, higher than the rate of students who listened to the failure-idol story, which is 0.2916. Similarly, the average growth rate of entrepreneurial intention for students who listened to the successful-peer story is 1.1045, higher than the rate of students who listened to the successful-idol story, which is 0.4256.

Experiment 2 tested the moderating effect of distance between students and entrepreneurial role models, where distance is measured by variance. Results in Appendix Table A4 show that an entrepreneurial role model (*F* = 13.145, *P* < 0.05) has a significant impact on the students’ entrepreneurial intention as post-experiment students’ entrepreneurial intention (3.8387) is higher than students’ original entrepreneurial intention (3.2247). The interactions between the entrepreneurial stories and moderating variables have a significant impact on the students’ entrepreneurial intention (*F* = 4.029, *p* < 0.05), thus, proving that the distance between student and the role model is the moderating variable between the role model story and the entrepreneurial intention and supporting hypothesis 5.

Before experiment 2, the average numbers of entrepreneurial intention for students in the peer and idol groups were very close. But after entering different groups, the average number of students’ entrepreneurial intention in the peers group increased more than the average number of entrepreneurial intention of all students. The growth rate for students in idol group is smaller than students in peers group. Therefore, the distance between students and entrepreneurial role models has a negative effect on the relationship between entrepreneurial stories and students’ entrepreneurial intention. The closer the relationship is, the more positive effect between the two variables. In summary, hypothesis 6 is supported.

In summary, the above two experiments prove all hypotheses.

## Discussion

This study investigates the effect of storytelling on individuals’ entrepreneurial intention. Results show that both successful stories and failure stories positively affect individuals’ perceived attitudes. Furthermore, rather than the idol stories which are commonly utilized by educators in entrepreneurship education, peer stories are more effective in arousing individuals’ entrepreneurial intention. This result complies with Hite and Hesterly’s (2001) proposition that, the closer the entrepreneurial model is, the greater the impact on individuals. It reveals that the distance between audience and role models will decrease the storytelling’s persuasiveness. These results highlight that the effects of storytelling vary in relation with different role models. We consider that it is a potential path to optimize EEPs in the future. Educational content of EEPs should be examined cautiously and utilized according to the particular teaching aim, regardless of whether the contents are popular or not.

In addition, our results indicate that individuals’ self-efficacy moderates the relationship between entrepreneurial storytelling and entrepreneurial intention. In other words, role model stories are more likely to arouse entrepreneurial intentions for individuals who possess higher self-efficacy. In line with Bandura’s theory of self-efficacy, our study shows that individuals with a high sense of self-efficacy often expect more future successes. One explanation is that individuals with high self-efficacy are more sensitive to positive outcomes and neglect the negative signals such as potential problems or risks of failure. Thus, successful stories are more likely to inspire individuals with high self-efficacy. By synthesizing, the current study contributes to entrepreneurship education by providing practical implications for Case-based teaching.

### Limitations

First, considering our research topic, the sample size is relatively small. All students are from the same university and the homogeneity of samples may influence the experimental results. A huge sample size with diverse backgrounds may reduce such influences. Second, freshmen are not the best choice for subjects as they do not face career choices immediately, and starting a business is not a realistic option for them. Thus, relevant empirical researches are needed to test the validity. Third, although we have made great efforts, it difficult to ensure the control groups are really “equivalent” to each other. For instance, even teachers in the experiments give the same story, but the way they describe the story and express themselves creates differences. To avoid or reduce such potential systemic biases, we rehearsed the experiment cautiously, from model stories to declarative languages. Fourth, this study merely predicts entrepreneurial intentions rather than entrepreneurial behaviors. This limitation widely exists in entrepreneurship research as it’s very difficult to predict and measure entrepreneurial behaviors. We intend to follow up this sample in a few years, if possible.

## Conclusion

This research aims to better understand the storytelling in entrepreneurship education. Many previous studies which mentioned the impact of storytelling as role model stories are widely utilized in EEPs. However, many of them treat the storytelling in entrepreneurship education as an undifferentiated whole. Only a few scholars have tried to tease out the distinctive effect from different types of entrepreneurial stories. Within the frame work of our study, we find distinctive educational content influences audiences differently through individuals’ entrepreneurial passions. Counterintuitively, rather than idol stories, peer stories are more inclined to increase individuals’ entrepreneurial intentions. Although many teachers have spent a lot of time and energy writing biographies for famous idols and telling these stories in their classes, our research shows that such efforts may not reach their expectations. To a certain extent, this insight is coherent with some research results from different fields like health (Lockwood et al., 2004; Hindle and Klyver, 2007; Adekiya and Ibrahim, 2016). Thus, our study provides some theoretical and practical implications for educators who engage in entrepreneurship education.

In addition, we investigated storytelling’s influence process by testing the mediating effect of entrepreneurial passion and the moderating effect of self-efficacy. Educators can invoke this model to better understand the teaching mechanism, and thus update their training programs. As noted earlier, individuals with low self-efficacy are less motivated by storytelling. This suggests that in our teaching or training, we should pay attention to students with low self-efficacy, increasing their self-efficacy perception or utilizing more persuasive role models. Such activities will strengthen case studies in the teaching process.

This study advances our knowledge about entrepreneurship education and provides an important foundation for future research. Both contents and methods are important factors for entrepreneurship teaching. More future research is needed to explore the relationship between entrepreneurship education and entrepreneurial behavior. We suggest that entrepreneurship education should not only promote individuals’ intention but find the right person who needs it. For instance, we should not overemphasize entrepreneurial success for individuals with high self-efficacy in case they are overconfident, but encourage individuals who possess low self-efficacy. A significant amount of research into entrepreneurship education is necessary to better understand the teaching mechanism, process and influences.

## Ethics Statement

This study was reviewed and approved by the Ethics Committee of Southwest Jiatong university. This study was carried out in accordance with the recommendations of the Ethics Committee of Academic Committee at the Southwest Jiaotong University with informed consent from all participates. All participates gave written informed consent in accordance with the Declaration of Helsinki. The protocol was approved by the Ethics Committee of Academic Committee.

## Author Contributions

FL, JM, and RL participated in the design of this study and performed the statistical analysis and carried out the study and collected important background information. RL drafted the manuscript. FL, JM, and RL carried out the concepts, design, definition of intellectual content, literature search, data acquisition, data analysis, and manuscript preparation. All authors read and approved the final manuscript.

## Conflict of Interest Statement

The authors declare that the research was conducted in the absence of any commercial or financial relationships that could be construed as a potential conflict of interest.

## Footnotes

1 Data Resource: GEM (2014), TEA: includes the business that operate for less than three and a half years.

## Supplementary Material

DATA SHEET S1 Original data.

## APPENDIX

**Table A1:** Experiment 1: Descriptive statistics and correlations.

Variables	Mean	*SD*	1	2	3	4	5	6	7
(1) Entrepreneurial role model stories	0.50	0.503							
(2) Gender	0.50	0.503	0.081						
(3) Self-efficacy (T1)	3.2534	0.72064	0.207	-0.002					
(4) Entrepreneurial intention (T1)	2.9196	0.87072	-0.029	0.023	0.066				
Successful stories EI (T1)	2.8946								
Failure stories EI (TI)	2.9447								
(5) Entrepreneurial intention (T2)	3.8678	0.70648	0.511**	0.335**	0.287*	-0.085			.
Successful stories EI (T2)	4.2265								
Failure stories EI (T2)	3.5091								
(6) Self-efficacy (T2)	3.9845	0.96640	0.218	0.154	0.01	-0.568	0.566		
(7) Entrepreneurial passion	4.4126	0.79782	0.527**	0.194	0.251*	-0.074	0.542**	0.425**	1
Successful stories EP	4.9183								
Failure stories EP	3.9069								

*^∗^*p* < 0.05, ^∗∗^*p* < 0.01.*

**Table A2:** Experiment 1: The regression results of hierarchical linear regression.

	Standardized coefficients		
	
	Model 1	Model 2	Model 3
**Control variables**			
Gender (1 = female)	0.338**	0.211**	0.132
Self-efficacy (T1)	0.296**	0.197*	0.126
Entrepreneurial intention (T1)	-0.112	0.249**	0.222*
Successful stories EI (T1)			
Failure stories EI (TI)			
**Main effects**			
Entrepreneurial role model stories		0.330**	0.191*
Self-efficacy (T2)		0.601**	0.406**
**Interaction effect**			
Entrepreneurial role model stories × self-efficacy			0.388**
***R*^2^**	0.174**	0.586**	0.651**

*^∗^p < 0.05, ^∗∗^p < 0.01. N = 74.*

**Table A3:** Experiment 1: The mediating effects of entrepreneurial passion.

	Independent variable	Dependent variable	Standardized Coefficients	Sig.
Model 1	ERMS	EI (T2)	0.511	0.000
Model 2	ERMS	EP	0.527	0.000
Model 3	EP	EI (T2)	0.542	0.000
Model 4	EP	EI (T2)	0.377	0.001
	ERMS		0.313	0.006

*ERMS, entrepreneurial role model stories; EP, entrepreneurial passion; EI, entrepreneurial intention.*

**Table A4:** Experiment 2: The results of descriptive statistics and variance analysis.

	Description statistic		Variance analysis	
		
Variable	Mean (T1EI)	Mean (T2EI)	F	Sig
ERMS			13.145	0.001
ERMS (Successful)	3.2846	4.0497 (0.7651)		
ERMS (failure)	3.1648	3.6276 (0.4628)		
DRMA			9.277	0.003
Idol	3.2288	3.5875 (0.3587)		
Peer	3.2206	4.0899 (0.8693)		
ERMS × DRMA			4.029	0.048
Successful × idol	3.3288	3.6594 (0.3306)		
Successful × peer	3.3355	4.4400 (1.1045)		
Failure × idol	3.2239	3.5515 (0.3276)		
Failure × peer	3.1059	3.7398 (0.6339)		

*DRMA, the distance between role model and audience; ERMS, entrepreneurial role model stories.*

### Variable Measurement

All variable was measured by asking respondents to rate themselves.

1 means “Totally disagree” and 7 means “Totally agree.”

#### Entrepreneurial Intention

(1) How interested they were in setting up their own business.
(2) To what extent they had considered setting up their own business.
(3) To what extent they had been preparing to set up their own business.
(4) How likely it was that they were going to try hard to set up their own business.

#### Entrepreneurial Passion

(1) Establishing a new company excites me.
(2) Owning my own company energizes me.
(3) Nurturing a new business through its emerging success is enjoyable.

#### Self-Efficacy

I am:

(1) Being able to solve problems.
(2) Managing money.
(3) Being creative.
(4) Getting people to agree with you.
(5) Being a leader.
(6) Making decisions.
